# Unique osteological evidence for human-animal gladiatorial combat in Roman Britain

**DOI:** 10.1371/journal.pone.0319847

**Published:** 2025-04-23

**Authors:** T. J. U. Thompson, D. Errickson, Christine McDonnell, Malin Holst, Anwen Caffell, John Pearce, Rebecca L. Gowland

**Affiliations:** 1 Department of Anthropology, Maynooth University, Maynooth, Ireland; 2 Cranfield’s Forensic Institute, Cranfield University, Bedford, United Kingdom; 3 York Archaeological Trust, York, United Kingdom; 4 York Osteoarchaeology Ltd, York, United Kingdom; 5 Department of Archaeology, University of York, York, United Kingdom; 6 Department of Archaeology, Durham University, Durham, United Kingdom; 7 Department of Classics, King’s College London, London, United Kingdom; University of Bern, Institute of Forensic Medicine, SWITZERLAND

## Abstract

The spectacle of Roman gladiatorial combat captures the public imagination and elicits significant scholarly interest. Skeletal evidence associated with gladiatorial combat is rare, with most evidence deriving from written or visual sources. A single skeleton from a Roman cemetery outside of York where gladiators arguably were buried presented with unusual lesions. Investigation, including comparative work from modern zoological institutions, has demonstrated that these marks originate from large cat scavenging. Thus, we present the first physical evidence for human-animal gladiatorial combat from the Roman period seen anywhere in Europe.

## Introduction

The spectacle of Roman gladiatorial combat captures the public imagination and elicits significant scholarly interest. There is a wealth of archaeological evidence for the presence of gladiators and gladiatorial combat from across the Roman Empire in the form of amphitheatres and visual representations of arena combat, as well as training complexes, and occasionally the bodily remains of the gladiators themselves [1–5]. The most direct and unequivocal evidence for gladiatorial combat is trauma on the skeletons of those believed to have been gladiators. Such evidence is, however, surprisingly limited. The best example is the gladiator cemetery at Ephesus, in which a minimum of 68 individuals were excavated. Eleven of these bodies showed evidence of well-healed antemortem cranial trauma, while ten had cranial injuries incurring around the time of death [3]. These injuries were produced through a combination of blunt and sharp force trauma, and all were consistent with the type of interpersonal combat described or depicted to have taken place within the gladiatorial arena (e.g.,: [3]). Further bioarchaeological analysis of the skeletal remains noted a wheat and barley-rich diet, as well as tentative identification of the use of a plant ash beverage recorded in texts to have been imbibed by gladiators [6]. In London, [5]’s analysis of Roman-period human skeletal fragments from Lower Walbrook also identified a high prevalence of healed ante-mortem and peri-mortem fractures, including blunt force blows to the craniofacial bones and sharp-force injuries to the cranium. Although the burial context for these individuals has not specifically been linked to gladiators, the authors note some striking parallels in the type and location of the injuries with those excavated from Ephesus. Other bioarchaeological evidence includes disarticulated bones of putative gladiators adjacent to an amphitheatre in Trier, Germany, and a possible example of an individual from Pompeii [5,7].

**Fig 1:**
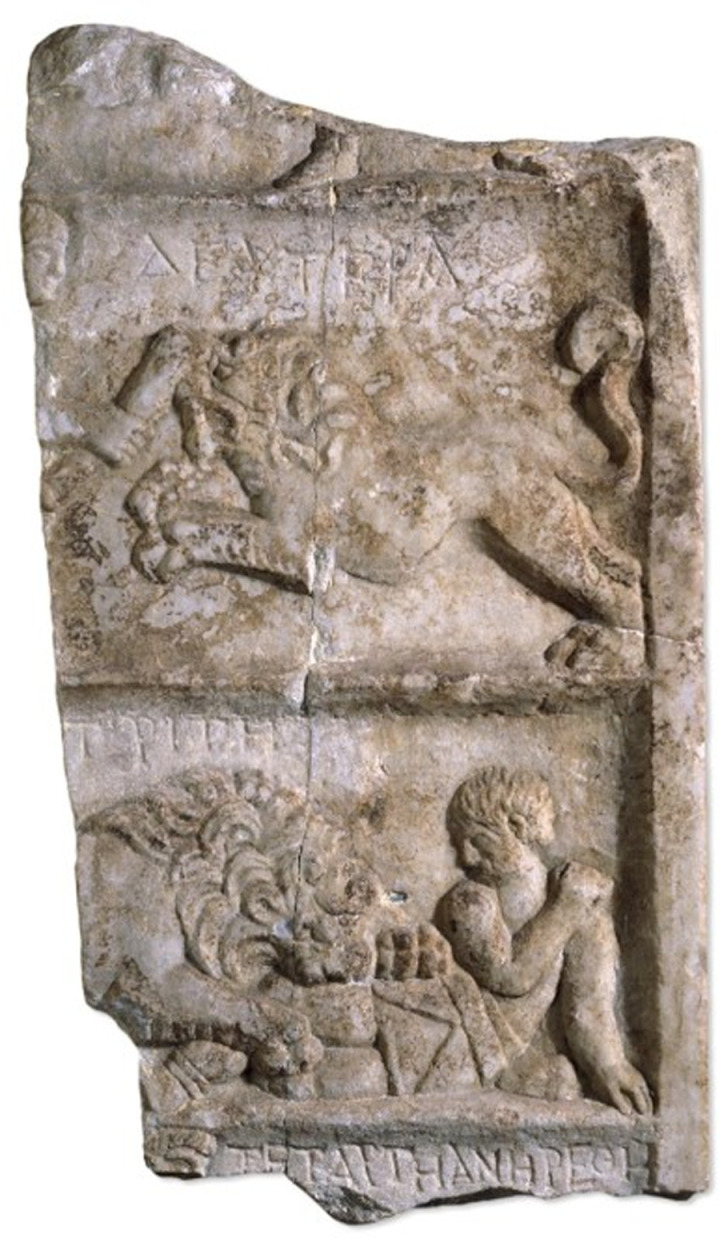
Marble relief with lion and gladiator [© The Trustees of the British Museum. Shared under a Creative Commons Attribution-NonCommercial-ShareAlike 4.0 International (CC BY-NC-SA 4.0) licence].

In addition to person-to-person combat, Roman amphitheatres also staged ‘beast hunts’ (*venationes*), which pitched people against animals (Fig 1), a spectacle lasting from the Republican period until late antiquity*.* Such spectacles at Rome are extensively documented in textual evidence; beyond the metropolis, inscriptions, mosaics and monumental stone sculpture commemorate similar events as well as the generosity of patrons which underpinned them [2,8,9]. The familiarity of the spectacle to Roman audiences is seen in its common reproduction on mass-produced ceramics [10,11]. In these ‘beast hunts’, trained performers (‘*venatores*’) were armed and placed in an arena to ‘hunt’ large cats (including lions, tigers and leopards), bears, or large herbivores (including elephants and wild boar as well as stags and bulls). Animals were used, too, as the agents of spectacular mutilation and execution of criminals, captives from warfare and other perceived deviants, including Christians, who were also sometimes forced to participate in such events, known as *‘damnatio ad bestias*’ [12]. These would sometimes include the re-enactment of mythical narratives as executions [13].

The nature and scale of person/animal combat in Roman Britain is contested. While images survive of such confrontations, there has been no published evidence to date which provides direct testimony of such events taking place in the province. The same is true of much of non-Mediterranean Europe in the Roman period, which lends a wider context to the results of this study. Here, we evaluate lesions identified on a skeleton excavated from Driffield Terrace, York, England, a site putatively considered a burial ground for gladiatorial combatants (see below). The lesions are consistent with large animal bite marks. We contextualise and evaluate this evidence in relation to the historical and epigraphic data, as well as zoological analysis of bite marks from a range of animals.

## Materials

### Driffield Terrace cemetery, York

Driffield Terrace is situated approximately 1km to the south west of York city centre. York (Roman Eboracum) was founded as a legionary fortress by the 9th legion which was replaced by the 6th legion sometime before the year 120 AD and who were garrisoned there until the end of the Roman period in the early fifth century [14]. Burial within settlements was forbidden in the Roman period, and the dead were, instead, often buried alongside the major roads leading to and from urban areas. Consistent with this, the Driffield Terrace burial ground is located along the major routeway between York and London, where it intersects with roads running from the west.

In 2004, proposed development of Driffield Terrace instigated an archaeological evaluation, revealing the presence of human burials and the need for a full excavation, which took place between late 2004 and early 2005. A total of 59 inhumations and 13 cremation burials were recovered from the initial area (Number 3, Driffield Terrace), and further excavations at another part of Driffield Terrace (Number 6) revealed an additional 23 inhumations and one cremation burial. Some disarticulated human remains were also recovered. The burials range in date from the first or early second centuries AD to the late fourth century AD [15].

An exceptional feature of this cemetery was the very high proportion of decapitation burials (approximately 70% of those with sufficient preservation to ascertain this [16]). Decapitation burials are a well-known phenomenon within Roman Britain, with decapitation mostly considered a post-mortem burial ritual, although this is debated [17–19]. The frequency of such burials is usually placed at around 5 to 6% of all burials [20], but this is sometimes higher in rural cemeteries (e.g., 14% at Kempston, Bedfordshire) [21,22]. In the Roman period, all ages and both sexes were accorded this rite, with the removal of the head usually accomplished through cuts delivered from the front to back of the neck [21]. Another exceptional aspect of the Driffield Terrace burials is that the majority of the decapitations occurred from back to front, a manner more usually associated with execution [16,22]. A further anomaly was that the head was not always completely removed, suggesting that the primary aim was to execute the person, rather than to conduct a funerary ritual. Here, as elsewhere in Roman Britain, the decapitated head was not placed in the anatomical position, but typically by the feet and legs. Finally, the demographic analysis showed that, with the exception of a single female and seven non-adults, all of those excavated were males aged between 18-45 years [16]. While a male bias has been reported for some cemeteries in Roman Britain and might be expected at a site where the military was garrisoned (e.g., York), such a strong bias is exceptional [23].

The majority of the inhumations were single, supine burials, very few of which had any accompanying grave goods. There were five double burials, one triple burial and one grave containing four bodies. The graves were shallow and there was no evidence of grave markers, leading to some intercutting [24]. Evidence of coffins was present most frequently in one area of the cemetery (6 Driffield Terrace), along with some evidence for hobnail boots. None of these funerary features suggest a high status, although the lack of grave goods conforms in general to contemporary ritual norms [25]. One exceptional burial was individual SK37, from the area of 3 Driffield Terrace, who was wearing heavy iron rings around his ankles, thought likely to have been present during life rather than a feature of his death alone (see [22] for a detailed discussion of this individual).

Pathological analysis of the skeletons revealed a number of unusual features. In addition to the extensive peri-mortem sharp force trauma associated with the decapitations, there was a high prevalence of healed or healing ante-mortem trauma. The location and type of injuries, including healed cranio-facial fractures, fractured teeth, fractured right first metacarpals and vertebrae, are those strongly associated with interpersonal violence and typical of injury recidivists [16,22,26].

A number of interpretations of this unusual site have been suggested, including that the burials were victims of a massacre by Emperor Caracalla in AD 211, purging his enemies after accession [22,24]. This has been discounted, however, as the burials accumulated over centuries, rather than following a single catastrophic event. Another interpretation is that the site was an execution cemetery, or a burial ground exclusively for soldiers, but these explanations are not consistent with the palaeopathological evidence [24].

Some aspects of the healed cranial trauma are consistent with the ante-mortem injuries identified at Ephesus, the known gladiator burial ground discussed above. Indeed, the individuals from Driffield Terrace exhibited a higher prevalence of healed lesions even than those of Ephesus (23% of individuals at Driffield Terrace, compared to 16% at Ephesus; [16]). The vast majority of injuries at both Driffield Terrace and Ephesus were located on the left side of the cranium and most were on the frontal bone, common in face-to-face interpersonal combat with a right handed aggressor.

Finally, the isotopic analyses of individuals excavated from Driffield Terrace are also atypical when compared to other burial grounds, showing a much wider range of values, in terms of diet and likely geographical origins [22,27]. There has been some minor reinterpretation of local versus overseas origins over time as the range of isotope values known to be feasibly derived from Britain has been modified slightly in recent years. Analysis by [28] of seven individuals from Driffield Terrace identified the presence of a person with close genetic affinities with the Middle East and this was supported by the isotopic evidence. More recent work has identified Scandinavian Peninsula-related ancestry in another individual from the same group [29].

Overall, the osteological evidence provides us with a picture of young or middle aged men, originating from across the Roman Empire, engaging in repetitive and sustained acts of violence. The skeletal evidence for trauma, together with the exceptional demography and decapitations, are consistent with death as a consequence of participation in a combat arena [24]. Some commentators on these burials remain cautious or sceptical concerning the identities of the men buried here, preferring a wider range of possibilities, also including captives, criminals and soldiers [14,27]. Full consideration of their identity would require a more detailed review than is possible here and in any case would remain inconclusive, in the absence of epigraphic evidence, or of many confidently attributed gladiator cemeteries against which the Driffield Terrace cemetery can be compared. On the one hand, if these individuals are gladiators, then their apparent execution is not straightforwardly compatible with the evidence for the method of dispatching injured gladiators at the end of a bout (throat-cutting), though this evidence is itself very limited and open to discussion [30]. On the other hand, the evidence of burial location and the recurring care for due funerary ritual makes it tempting to identify this as the resting place for a *familia gladiatoria* (troupe of gladiators), perhaps even one associated with the legion, which would provide the institutional continuity behind such a group. Such legionary *familiae* are occasionally attested [31]. An affiliation to the legion would also help explain the placing of this burial area in what is considered to be one of the most prestigious, on the highest point in the environs of the city and with a military connection attested in the presence of soliders’ tombstones [14].

All of the above provides useful context against which to assess the putative bite marks on the pelvis of skeleton 6DT19.

### Individual 6DT19

Individual 6DT19 was one of three adults deposited in a supine position in the same box in a SW-NE aligned grave (grave 1130), in the densely used burial space excavated at no. 6 Driffield Terrace (see [32] for further detail and photographs). The grave itself was cut within a possible earlier burial mound and its fill contained a mass of butchered and gnawed horse bone [24]. A third century date has been suggested based on stratigraphy for the phase in which this burial occurred, probably in the middle to later part of the century [15,24].

**Fig 2:**
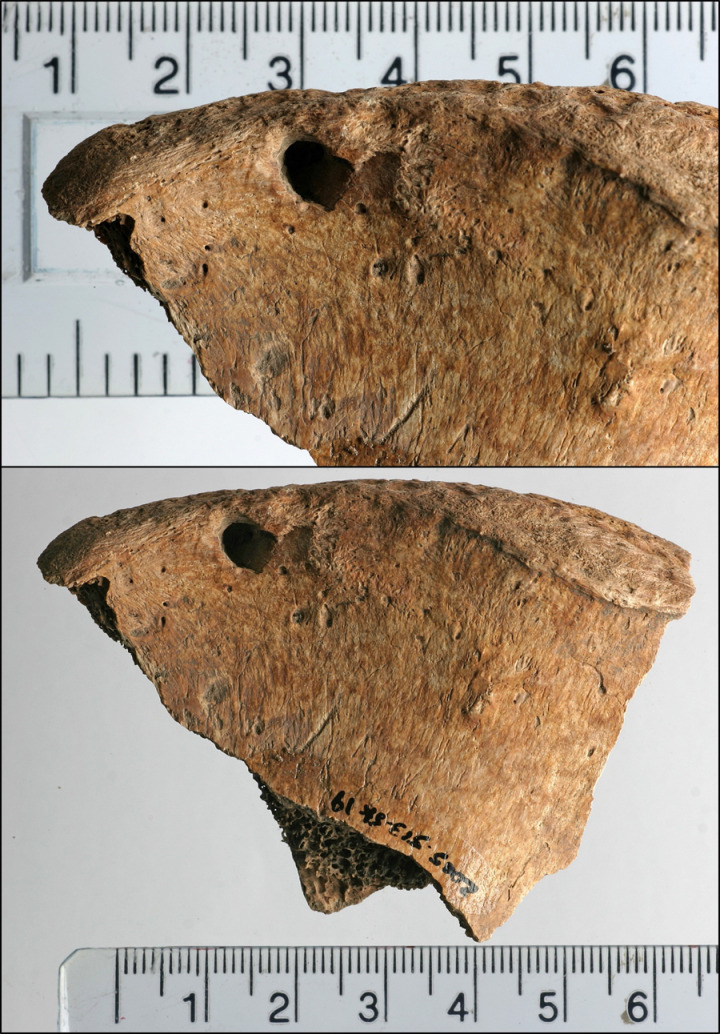
Lesions on the left iliac spine of 6DT19.

**Fig 3:**
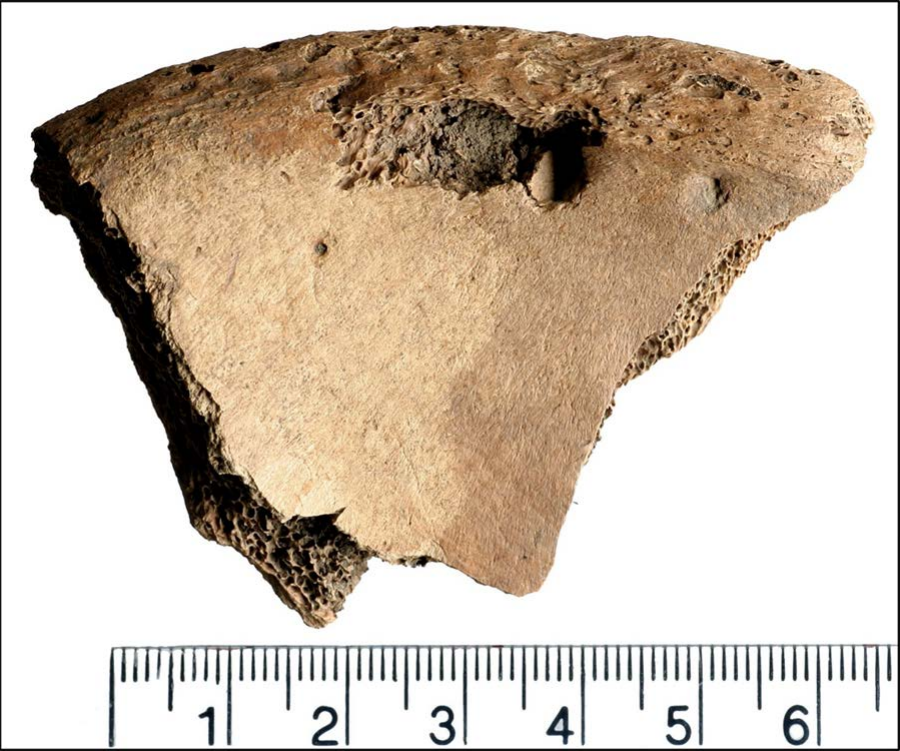
Lesion on the left iliac spine of 6DT19.

**Fig 4:**
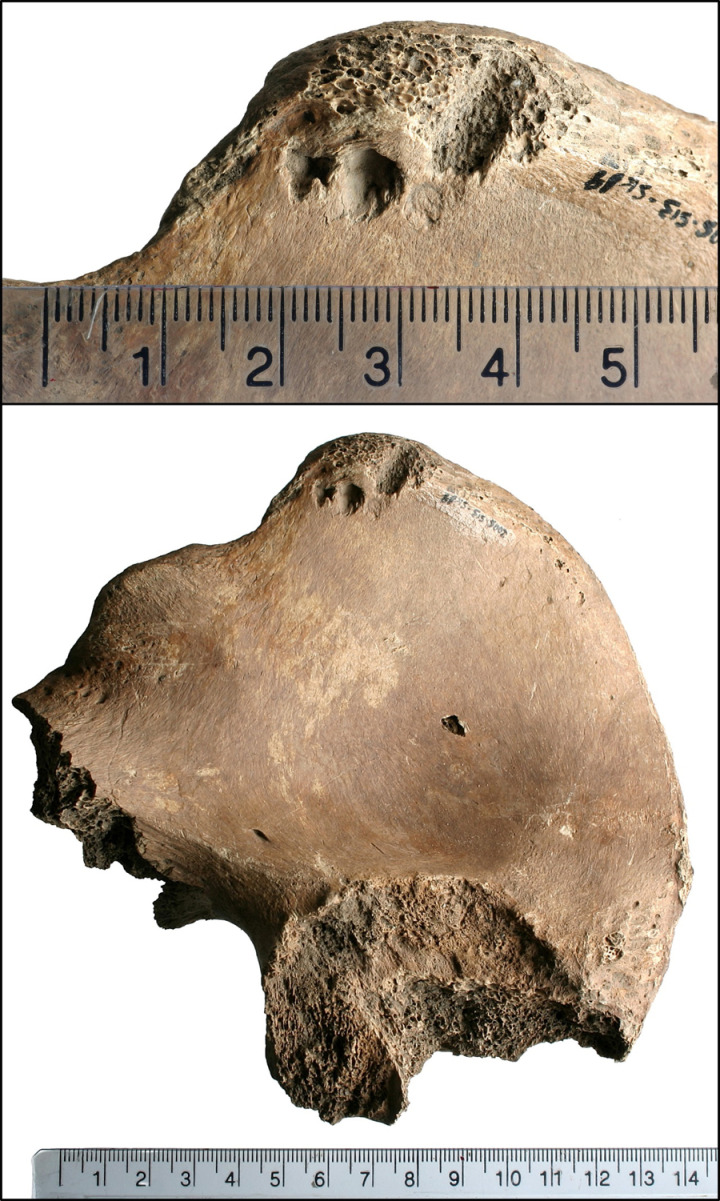
Lesion on the right ilium of 6DT19.

[16]’s bioarchaeological study of this individual recorded completeness of the skeleton at 70-80% with Good (Grade 2) preservation based on [33] standards. The analysis concluded that 6DT19 was a male aged 26 to 35 years and 171.9 cm ( ± 3.37 cm) tall. Further, their isotopic profile shows a higher delta 18 Oxygen Phosphate than would usually be expected in Britain, suggesting origins at a lower latitude and warmer climate, but this does still fall within the margins of what could be expected within the south and west of the country [27]. 6DT19 had been decapitated with a single cut between the second and third cervical vertebrae, delivered from behind. The decapitated head was placed in the normal anatomical position, facing upwards to the right. Additional peri-mortem trauma was present in the form of a series of small depressions on both sides of the pelvis, located close to the iliac crest and spines [16]. These are the parts of the pelvis that can be prominent in living people, and easily palpated just above the hips. Examples of the trauma are presented in Figs 2–4.

The left ilium had three discrete depressions on the anterior/medial surface of the iliac crest (Figs 2 and 3), including:

1. a small shallow indentation (2.8 mm in diameter) located ~ 20 mm posterior to the ASIS and ~ 6 mm inferior to the iliac crest
2. a deep depression (~6 mm in diameter, 4 mm deep) located ~ 33 mm posterior to the ASIS (8 mm posterior to depression 1), 6 mm inferior to the iliac crest
3. an indistinct shallow indentation (2 mm in diameter) just anterior to the midpoint of the iliac crest (~77 mm posterior to the ASIS, 10 mm inferior to the iliac crest).

There was a deep, roughly circular depression (6.5 mm in diameter, 5 mm deep) on the posterior/lateral surface, located ~ 25 mm posterior to the ASIS and 2.5 mm inferior to the iliac crest, and a second slight indentation (3 mm in diameter, 0.8 mm deep) located ~ 9.5 mm posterior to the ASIS. All depressions and indentations had small adhering flakes of bone pushed into the lesions.

The right ilium (blade of the pelvis) had a row of three indentations close together on the anterior/medial surface of the anterior superior iliac spine (ASIS) as shown in Fig 4. These included:

1. A small triangular lesion (4 × 4 mm, 1.5 mm deep) located ~ 4 mm posterior to the ASIS
2. A circular depression (5.2 × 7.3 mm, 2.7 mm deep) located 9.5 mm posterior to the ASIS and 9 mm inferior to the iliac crest
3. An indistinct shallow, roughly circular lesion (~3 mm in diameter) posterior to depression 2.

There were two shallow linear crushed areas on the posterior/lateral surface of the ilium, close to, and perpendicular to, the iliac crest. One was located ~ 22 mm posterior to the ASIS (13 mm long, 1.3 mm wide), and the second was located ~ 30 mm posterior to the ASIS (7 mm long, 1.5 mm wide).

[19] undertook an initial analysis of this person’s skeleton, concluding that these unusual peri-mortem depressions were likely carnivore bite marks. [16]’s subsequent analysis was more cautious, and whilst they did not disagree with [19]’s assessment, they suggested that the lesions required further assessment by a bite-mark specialist. Below we present this analysis and the results.

## Methods

Bite marks on soft and hard tissues can be associated with particular species of animal through analysis of characteristics such as depth, size, shape, position and location of the mark. Such research is well established and widely accepted [34–36] and requires the bite mark to be properly characterised and matched to the causal species. Traditional approaches to bite mark analysis have used photography but this does not allow for depth and volume measurements. Work using 3D surface light scanning methods have demonstrated their utility in recording and interpreting a variety of traumatic injuries [37–39].

With this in mind, Skeleton 6DT19 was re-examined for signs of injury and subsequently 3D documented using a non-contact structured light scanner. This allowed for a more detailed analysis of the shape and positioning of injuries as well as three-dimensional comparisons with the modern faunal comparators (see below). An HP 3D structured light scanner Pro S3 was used. The projector and camera were pointed down towards the pelvis with the fragments raised 8-10 cm from the bench and about 30-80 cm away from the bar. The camera was set at 1/30 exposure and the projection was set at 225 brightness. Calibration was at 120mm. The projector was set at 80mm on the bar and the camera was set at 200mm on the bar at a 27-degree angle. A manual scan sequence was used for the scanning process. Free contact pair selection was used to stitch the scans together to create a cohesive model. For fusion of the model, resolution was set at 2000, sharpness set at 1, and close holes set at 0%. This methodology follows the guidelines presented in [40,41].

**Fig 5:**
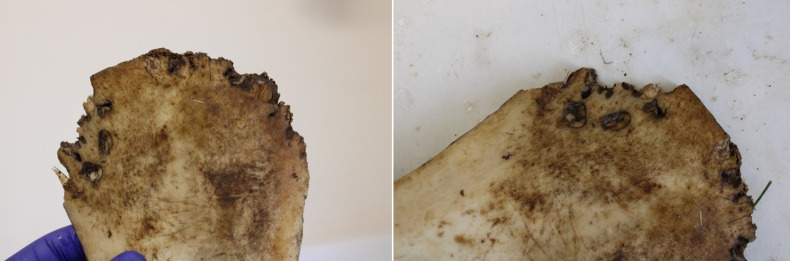
Puncture injuries by large felid scavenging on both sides of bone (from the research detailed in [35]).

**Fig 6:**
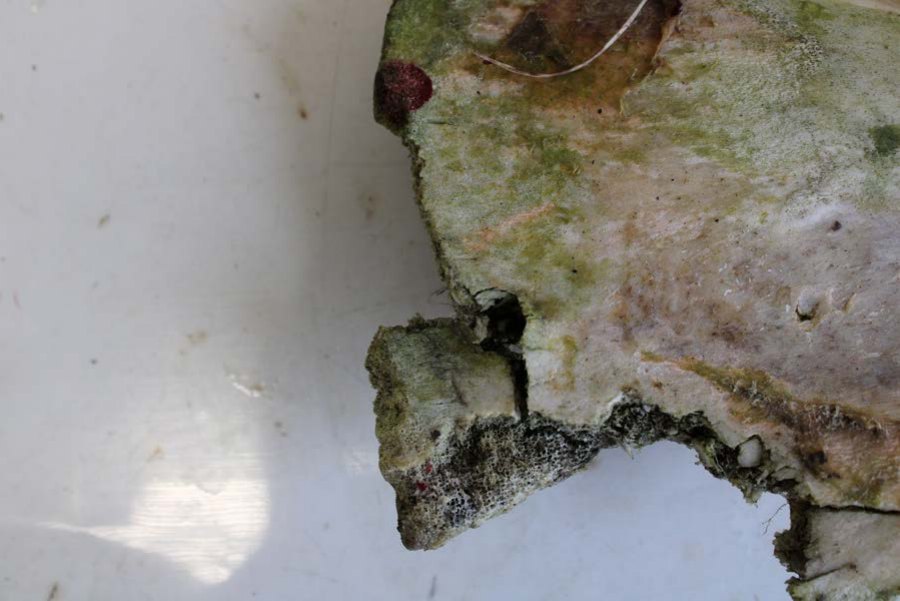
Puncture injury from leopard feeding (from the research detailed in [35]).

Primary research by [35] has provided a range of examples of bite and scavenging marks by felids on bone using controlled experiments on contemporary material from zoos in England (see also Figs 5 and 6). Amongst other animals, this work examined bite marks in fresh carcases from cheetahs, lions, tigers and leopards. Examination of the scavenging damage was performed using the same approach as described above and therefore allowed for the direct comparison and interpretation of the injuries experienced by Skeleton 6DT19.

Ethical Approval was obtained for this study (Cranfield University, CURES/7876/2019).

## Results and discussion

### Comparative bite mark analysis

Standard photography converts three-dimensional features into two-dimensional images and thus comparison of variables such as depth and volume can be limited. The results of the structured light surface scanning here (presented as Supplementary Data) allowed for the models to be measured and superimposed onto each other to permit a more accurate assessment of similarity and difference. Full discussion of this analytical and quantitative approach is outside the scope of this paper, but readers are directed to [37–39] for this detail.

Different species of large carnivore attack in different ways and much of the research which describes soft and hard tissue lesions resulting from animal attacks is provided in the clinical and forensic literature. Bite marks associated with post-mortem scavenging are also important to consider [36].

Deaths caused by lions and tigers tend to result from trauma to the neck area, involving the crushing of soft tissue structures and fracturing of the vertebrae, causing suffocation [42,43]. Both species use their weight to push down the victim, often also leaving extensive damage to the shoulders, arms and chest [43]. Species such as leopards and jaguars focus on the head by puncturing or crushing the skull. Indeed, occasional Roman images of *damnatio ad bestias* show considerable accuracy in this regard. Most show the animal springing (or poised to spring) towards its target but on the Domus Sollertiana mosaic from El Djem, Tunisia, a leopard is depicted clinging to the thigh and torso of its captive victim, grasping the head in its mouth ([44], Fig 13). The bite marks on 6DT19 are located on the pelvis rather than neck and upper body. Lions and tigers have also been seen to drag their prey away, often by the legs [43], but lions have also been recorded as causing significant damage to the pelvis of their prey [42]. Another Roman period image appears to show such a case: a lion biting the thigh of a wounded *venator* on a relief from a likely funerary monument in Ephesus; the inscription which serves as a caption for the scene reports the fighter’s death (BM 1873,0505.1 relief | British Museum). Further examples are noted by [45].

Large cats have been shown to create puncture wounds (with penetration up to 9 cm) and occasionally causing curved bite marks from their incisors ([43,46]; Fig 5 presents examples of cheetah scavenging and Fig 6 presents an example from a leopard, both from the research detailed in [35]). The depth of the bite mark is less than the length of the tooth due to the presence of overlying soft tissues. Similar features, including curvature of the bite mark within the bone, are seen in 6DT19, along with a shorter depth of wound than canine tooth length.

Canines present different patterns of bite marks when compared to large cats, and the resulting traumatic injuries are mainly restricted to the soft tissues [47]. Dogs tend to pull humans to the ground by attacking the limbs [48] and then bite and tear at the remains. Damage to pelvic skeletal structures during such attacks has rarely been recorded [47,48], although dogs can leave pits, scoring and punctures on bones that become exposed during the attack, or by penetrating through thinner bones [48,49]. Such injuries are not consistent with the lesions on 6DT19.

Bear attacks tend to involve paws and claws as well as biting [50]. In a predatory context, they are known to drag their prey away [50]. Bears rear up onto their hind legs when attacking before lunging and the weight and severity of the attack leads to significant skeletal injury across the body, focussing on the chest and back region. Injuries from bear attacks tend to be located on the head, neck and upper limbs, with death generally due to exsanguination [51–54]. Soft tissue trauma has been noted around the abdomen and inguinal regions [53] and limb injuries are rare, although upper limbs are more commonly damaged than lower [51].

Boars have also been recorded in gladiatorial contexts, but although they cause deep traumatic injuries to the hips and thighs [52], these are from their tusks and are not consistent with the range of bite marks exhibited in 6DT19.

It is proposed, based on the evidence from the archaeological, medical and forensic evidence, that the bite marks on 6DT19 derive from a large felid, such as a lion. The shape is entirely consistent with documented cases of large cat bite marks (such as those presented in [35,46]). The location solely on the pelvis suggests that they were not part of an attack *per se*, but rather the result of scavenging at around the time of death. The decapitation of this individual was likely either to put him out of his misery at the point of death, or for the sake of conforming to customary practice. Again, this is consistent with the literature.

### Other possible interpretations

Other possible explanations for the lesions exhibited by 6DT19 include peri-mortem penetrating weapon injuries or taphonomic damage but these are not convincing. For example, one interpretation of a cranial injury at Ephesus was penetration from a trident [3], but these lesions are much larger than those on 6DT19 and they exhibit bevelling which is absent in the lesions here. Arrow trauma could be a possibility, but [55]’s work examining penetrating projectile damage to bone demonstrated differently shaped wounds to those seen here. The characteristics and clustering of the lesions in the case of 6DT19 (for example, two shallow indentations and one deep depression anteriorly, and one deep circular indentation posteriorly) are more consistent with a carnivore bite mark than the isolated injury of a weapon. With regard the carnivores, the bite mark pattern is consistent with a large animal, likely a feline and are not canine for the reasons described above and in the referenced publications. However large canines such as a wolf (*Canis lupus*) should not be entirely ruled out as the literature for their characteristics expands. Taphonomic damage alone is also unlikely due to the appearance and margins of the lesions, which are the same colour as the surrounding bone (this differs if the break is post-mortem; [56]), and the adherence of bony fragments at the injury site (which occurs when soft tissue is present). These features are consistent with perimortem rather than postmortem injuries. Taken together, other possibilities have been dismissed.

### The bite marks and Roman spectacle culture

The most likely context for the trauma incurred by 6DT19 lies within Roman spectacle culture, the staging of often violent performances involving animals as combatants, as victims and as agents of execution, which likely accompanied other kinds of spectacle, primarily gladiatorial shows (see Fig 1) but also plays and chariot racing. In order to put the results of this study in context, the following paragraphs briefly sketch the nature of the surviving evidence for spectacles in Britain, emphasising its limitations, and identify the potential insights of this study for our understanding of spectacle in Roman York and beyond.

Structures to accommodate Roman-style spectacles, principally amphitheatres, but also theatres and at least one circus, are attested in Britain. In some cases, for example at Caerleon or Silchester, surviving examples indicate their original monumentality and the concomitant investment which underpinned them [10]. While spectacles could also be shown in general public spaces, such as the forum, it seems almost certain that York as the site of a legionary fortress and as a colony and provincial capital from the early third century AD would have possessed an amphitheatre, perhaps southwest of the fortress under King’s Manor [14].

Nevertheless, with occasional exceptions, such as evidence for tethering posts and pens for holding animals before their release into the arena (*carceres*), such structures give little direct clue to the specific performances which they accommodated [57]. Instead, images as well as inscriptions and artefacts provide the main evidence for the nature of spectacles in Roman Britain and for the performers who took part in them. Gladiators, animal fighters (*venatores* or *bestiarii*), charioteers and acrobats are represented on portable objects, above all ceramic and glass vessels as well the decorated handles of clasp knives, occasionally complemented by mosaics, wall paintings and possible tomb images. However, many of these objects are imported and/or present generic arena scenes, making it difficult to use them as direct witnesses to spectacle culture [2,10,58,59]. Among these, more weight can perhaps be placed on images created in Britain itself. For example, the diverse encounters rendered in barbotine on the Nene Valley and Colchester colour coated vessels of second and third century date suggests that *venationes*, i.e., combat between armed fighters and animals as well as other staged hunts and perhaps acrobatics involving animals, entertained audiences in the province’s arenas. In one case, showing fighters tormenting a bear, the labelling of combatants through captions suggests that a real scene is depicted [60]. The famous Venus mosaic from the villa at Rudston, East Yorkshire includes images of animals and possible hunters which again hint at the *venatio*. Even in these cases, however, the influence of external artistic inspiration (Gallic ceramics in the former case, North African mosaic traditions in the latter) frustrate their confident use as testimony to local spectacles ([61,62]; see further below). A recently excavated key handle from a late Roman town house in Leicester appears to show a lion attacking a human as a possible representation of *damnatio ad bestias*, the execution of criminals and captives by an animal agent, but its specific iconography is hard to parallel [63].

Reference has been made above to the trauma documented on human remains from Walbrook as likely evidence for gladiatorial spectacles [5]. The faunal remains documented from Britain which are potentially relevant to amphitheatre spectacles are very limited, as indeed they are more widely in the Roman empire [64,65]. The most recently published assemblage from a spectacle building, namely from the excavations of Chester’s amphitheatre, for example, closely resembles other legionary fortress animal bone assemblages, and gives no real insight into species involved in performance, though it hints at the food consumed by spectators [66]. Elsewhere in Britain a small number of cases have been identified as possible animal protagonists from the arena, drawn from the province’s own fauna: bear, boar, stag and bull [10,67,68]. In neighbouring provinces (Gaul and Germany) too there is as yet no trace among faunal assemblages of imported exotic animals, though an increasing quantity of evidence for the exploitation of bears ([69,70]; Lepetz, pers. comm.). At Viminacium, the site of a Roman legionary fortress near Kostolac in Serbia, the cut marks on bear bones show the consumption after spectacles of arena carcasses, a likely cause of the limited evidence so far documented among faunal assemblages for animals used in the arena [13]. The discovery of a leopard humerus and other forelimb bones associated with the amphitheatre at the same fortress is, at present, the only faunal evidence for the arena use of a big cat in Europe north of the Alps [71]. Even for Rome itself, some scepticism has been expressed about the mass slaughter of diverse exotic animals suggested by literary and visual sources. The meagre attestation of exotic wild animals in Mediterranean faunal remains assemblages prompts [64,65], for example, to doubt that North African felines in particular were ever shown in great numbers, despite the claims of the textual record [72]. Even in North Africa, the home of the key species favoured as the most prestigious arena protagonists, as well as at Rome, including the Colosseum, lions and leopards are only occasionally attested and are considerably outnumbered by bears in faunal assemblages. Other explanations for the cranial and phalangeal fragments which form most of the felid remains are possible, including the movement of pelts as trophies [73–75]. All of this is enough to say that a dependence on general analogies from literary evidence for understanding arena performances in provinces like Britain will misrepresent the variability in spectacle culture across the empire, almost certainly contingent on the availability of resources, local cultural preferences and the role of spectacle in urban social dynamics (e.g., [76] on the *venatio* in North Africa).

However, the identification of the animal bite as that of most likely a lion, now allows some wider conclusions to be drawn, for York, Britain and the wider northern Roman world. In York itself the arena remains unlocated. The city played multiple overlapping roles in the Roman period: legionary fortress, settlement agglomeration and then colony, residence for the provincial governor (after the division of Britannia in the early third century AD), and occasionally for the emperor himself [14]. Emperors, governors or local magistrates are all candidates for the impresario who placed a lion in a third century AD show as a *coup de (amphi)théâtre*.

The possible circumstances in which a lion or similar appeared as execution agent are various in the period to which the burial dates. In the context of wavering local loyalties, it has sometimes been argued that British amphitheatres saw exemplary punishment of mutinous soldiers in the third century AD, though the specific structural evidence invoked in support has not stood up to closer scrutiny [57,77]. Putting on *venationes* for soldiers’ enjoyment can also be conjectured. Such a scene may feature on a metal fitting of third century date, a probable scabbard mount, in which named detachments from two British legions stand before an animal group comprising a stag framed by a dog and a lion, as well as a peripheral dog-hare chase and a foregrounded pair of peacocks. The nature of the scene is disputed – [78] sees it as a Christian image, [79] as a ‘light-hearted medley’ - but it is tempting to identify the scene as a *venatio*, perhaps celebrating a joint achievement of the two legions featured acting in concord. Animal fighters perhaps formed part of the *familiae* (troupes) of gladiators attached to some individual legions; for example, *bestiarii* – another term for such combatants – are attested on papyri of late second or third century AD date documenting the *familia* connected to the fortress at Babylon, i.e., Old Cairo near Memphis [31].

Alternatively, evidence from York’s environs suggests that this spectacle is more plausibly placed in the context of municipal euergetism, i.e., the gift-giving culture which maintained the status of Roman urban elites. In the Humber estuary region, a cluster of villas connected to this class include late Roman mosaics depicting spectacles, mainly chariot racing. At one, Rudston in the Yorkshire Wolds, is a late third century AD mosaic which famously shows Venus at its centre, surrounded by armed figures and animals (lion, bull, leopard and stag). Two of the latter are captioned with possible stage names as ‘legendary’ performers - *Leo Flammefer*, the ‘fiery lion’ and *Taurus Omicida*, ‘Killer the bull’ [80]. Beside the bull is a motif – a crescent on the end of a stick - which resembles a badge used for the Telegenii, the most prominent of the corporations (*sodalitates*) of arena hunters who feature as performers in animal killing spectacles in North African mosaics [2]. The meaning of this mosaic is debated [61] but it is tempting, if unprovable, to argue from the bite mark evidence that it can now be more likely identified as a record of a *venatio* broadly contemporary with the burial, put on in York.

The North African echoes in the Rudston mosaic, both in the choice of scene and its details, have long been noted, the most recent assessment suggesting a patron or craftsman familiar with North African mosaics attempting to re-create a scene familiar from home, using mosaicists working in the regional traditions of north-east England [61]. The privileged connections between York and North Africa, linked to the Roman army, may help explain the presence of a lion in the city. These connections are most vividly seen in the presence in Eboracum in AD 208-11 of the emperor Septimius Severus and his sons, a dynasty originating in Leptis Magna in Libya, but they are also attested in ceramic evidence. [81] has recently re-examined and re-asserted older arguments for close connections between the legionary garrison at York and North Africa, notably those developed by [82]. The key evidence is ‘late Ebor ware’, a ceramic type made in the city in the third century AD, inspired by ceramic traditions in contemporary Tunisia and frequently occurring in assemblages around the legionary fortress. This provides likely testimony of potters linked to the Sixth legion being recruited from North Africa. The large number of North African amphorae in contemporary Eboracum, carrying oil from Tunisia, also illustrate the likely links in military supply between the city and Africa Proconsularis. As [81] argues, the context for this movement of specialist personnel and commodities may be the aftermath of the defeat of Clodius Albinus (AD 193), the former governor of Britain whose rebellion the legion had supported, with a change in legionary personnel and the promotion of their wider North African clientele by Septimius Severus as emperor.

Whatever the specific context, as witness to the long-distance movement northwards from the Mediterranean of *ferae africanae* (a Roman term for lions and leopards shown in the arena), the bite-mark evidence has significant logistical implications. The practicalities of wild animal movement, boxes and cages, ships and wagons, draught animals and foodstuffs on the hoof, animal keepers and trainers, all imply the existence in northern Europe, however occasional or intermittent, of the cavalcades documented in the Mediterranean for the transport of animals to Rome. *Sodalitates* (corporations) of animal hunters like the Telegeni, attested in Tunisia, make for plausible sources of experienced personnel, to take responsibility for animals in transit and mitigate their risk of death *en route* [65,83]. The famous animal capture mosaic from Piazza Armerina, Sicily, vividly conveys the beginnings of one such expedition [2].

Long-distance movement of this kind, with all its corollaries, need not characterise York alone as a special case. Just as the bite mark evidence allows more confident claiming of animal spectacle images from Britain such as the Rudston mosaic or Leicester key handle as representations of real events, so too may it permit the same for the more abundant and impressive visual evidence from neighbouring provinces. Mosaics as well as frescoes from the Rhineland and eastern Gaul, clustered along the key transport arteries that connected the Mediterranean to northern Europe, present well-observed images of *ferae africanae*. These mosaics are less well known than those of North Africa but nonetheless present memorable animal encounters [2]. For example, on a third century mosaic from a villa at Nennig, near Trier, Germany, in separate medallions a tiger fights an onager, a leopard is mortally wounded by a spear-armed *venator* and a lion is led away by a trainer, alongside bears fighting padded tormentors, as well as gladiators and fighters with cudgels, while the organ plays on ([84], Taf. 36-39). On a townhouse mosaic from Reims (the ‘mosaique des Hautes Promenades’, destroyed during World War I) many of the 35 panels feature single big cats, again including lions and leopards ([85], no. 38; [86]). Arguably the most spectacular representations however are in paint, the large-format (*megalographeia* or near life-size) frescoes of a tiger and leopard featuring in *venationes* painted in the late second or early third century walls in a room of a wealthy Roman *domus* (elite house) in the Domviertel, Cologne. These are among the most accomplished frescoes from Rome’s northern provinces ([87], Taf. VII, Abb. 74). Moving east, painted skins of leopards and tigers are rendered on the frescoes decorating the arena wall of the stone phase (mid second century) amphitheatre itself at Viminacium on the Danube. The discovery of leopard bones in the same arena noted above suggests that this was not a simple replication of a conventional scene but spoke directly to animals seen there [71]. From the Danube too comes an exceptional witness to the transport of big cats to northern Europe. Context demands caution in believing the curious incident of an abortive lion sacrifice at Carnuntum near Vienna in AD 167/8. The throwing of two lions into the Danube, required by a prophecy to bring a victorious end to the Marcomannic wars of Marcus Aurelius, went absurdly wrong; the lions swam away rather than drowning but died shortly afterwards. Heading for the non-Roman shore, they were bludgeoned to death on landing by the fearful inhabitants [88]. On the argument presented here, this episode becomes a little less absurd or implausible, at least the expectation that lions would be available for sacrifice at Carnuntum, where the degree of investment in spectacles is evidenced by a recently discovered school for gladiatorial training [4]. One lion bite does not fatally weaken arguments that mosaics and frescoes, still the key evidence for animal spectaculars, were meant to conjure up an otherworld of *voluptas* (‘pleasurable luxury’) in the affluent domestic spaces that housed them, rather than the realities of some local show [89]. However, it does suggest that sometimes at least such images would have prompted recollection of real human-animal confrontation and death in a local arena.

## Conclusions

The analysis of the lesions on skeleton 6DT19 provides convincing evidence that these were produced from the teeth of a large cat, such as a lion. Whether the trauma inflicted happened as part of a show or an execution, this evidence from York also carries several further potential insights. As key new direct testimony, the trauma markers from the Driffield Terrace cemetery therefore allows the spectacle culture of a city, in this case the colony and garrison community at York, and a province to be characterised with greater potential specificity, strengthening interpretations based on the uncertain foundations of visual images. It contributes to the understanding of a key phenomenon in Britain, i.e., the manipulation of spectacles by civic elites for urban populations, using such occasions to showcase their virtue, for example, generosity towards their fellow citizens or loyalty to the emperor through games celebrating the imperial cult. In a city such as York, the emperor himself is not to be excluded as a giver of games, either directly or through the provincial governor on his behalf, counting on the lion’s presence, whether in a show or exemplary execution, to imprint a memory of authority as well as munificence.

Documenting the provision of arena fauna with greater confidence enriches our understanding of the exploitation of wild species in the arena and contributes to modelling the environmental impact of spectacles, as well as their economic dimensions [90,91]. The logistical requirements of transporting arena animals to York, in the context of wider emerging evidence for animal movement [17] have implications for understanding the city’s connections to its northern British hinterland and potentially the wider Roman world. The consequences of the demand for animal shows in Rome on the capture of animals across the empire and their movement to the metropolis has been much discussed in recent years. It has been argued that emperors were almost as concerned with the supply of sufficient animals to populate *venationes* and other spectacles as they were for ensuring the availability of staple foodstuffs, with concomitant investment in infrastructure to sustain that supply [92]. As the bite marks reveal, not all this animal movement was centripetal; for places like York, nodes on the major transport axes, trafficked animals also headed to the imperial peripheries, especially where local connections to the army or emperor overrode potential cost or logistical obstacles.

By adopting a multidisciplinary approach to the analysis and interpretation of this individual’s final moments, we are able to further underline the importance of the osteological evidence in understanding the lives and deaths of those in the Roman past. Further, the potential of modern forensic studies to provide unique experimental evidence to explain the context of death in the past is demonstrated.

## Supporting information

S1 File Pelvis Model. (ZIP)
